# Supplementary figures and images for: Polarized Light Microscopy-Based Quantification of Scleral Collagen Fiber Bundle Remodeling in the Lens-Induced Myopia Mouse Model
Source: Life (Basel). 2025 Nov 13;15(11):1743. doi: 10.3390/life15111743 (PMC12653731; doi:10.3390/life15111743)

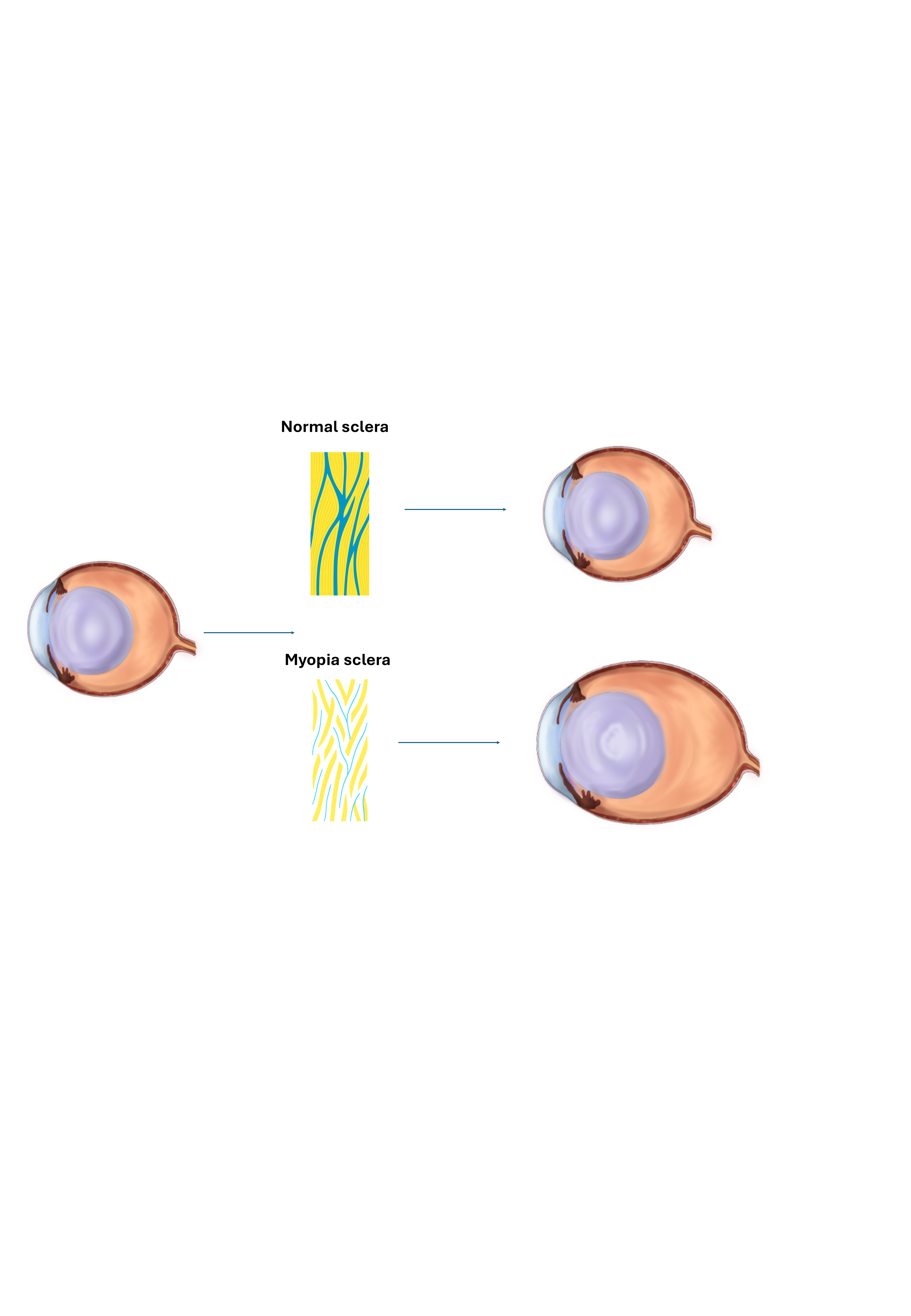

Supplement: Supplementary file 1 [file life-15-01743-s001.zip › Supplementary Fig.S1.TIF]
